# Apoptosis and Brain Dervived Neurotrophic Factor are increased in cortical neurons of Marfan Syndrome mice

**DOI:** 10.17912/micropub.biology.001651

**Published:** 2025-08-14

**Authors:** Mitra Esfandiarei, Faizan Anwar, Manogna Nuthi, Alisha Harrison, Mary Eunice Barrameda, Tala Curry-Koski, Kasey Pull, Theresa Currier Thomas, Nafisa Jadavji

**Affiliations:** 1 Biomedical Sciences, Midwestern University, Downers Grove, Illinois, United States; 2 Department of Basic Medical Sciences, University of Arizona, Tucson, Arizona, United States; 3 Department of Anesthesiology, Pharmacology, & Therapeutics, University of British Columbia, Vancouver, British Columbia, Canada; 4 Child Health, University of Arizona, Tucson, Arizona, United States; 5 Biomedical Sciences, Southern Illinois University Carbondale, Carbondale, Illinois, United States; 6 Neuroscience, Carleton University, Ottawa, Ontario, Canada

## Abstract

Marfan Syndrome (MFS) is an autosomal dominant genetic disorder that affects connective tissue throughout the body due to mutations in the fibrillin-1 ( *FBN1)* gene. There is a gap in our understanding of the impact of monogenic connective tissue aberrations on the brain. This study aimed to determine the impact of MFS on neurodegeneration in the cortical brain tissue of mice. We report increased levels of apoptosis in neurons within the sensory and motor cortical areas of female MFS mice. Additionally, we also report increased levels of neuronal plasticity in cortical brain tissue of both male and female MFS mice.

**Figure 1. Assessment of apoptosis and plasticity in Marfan Syndrome in motor and sensory motor areas:**
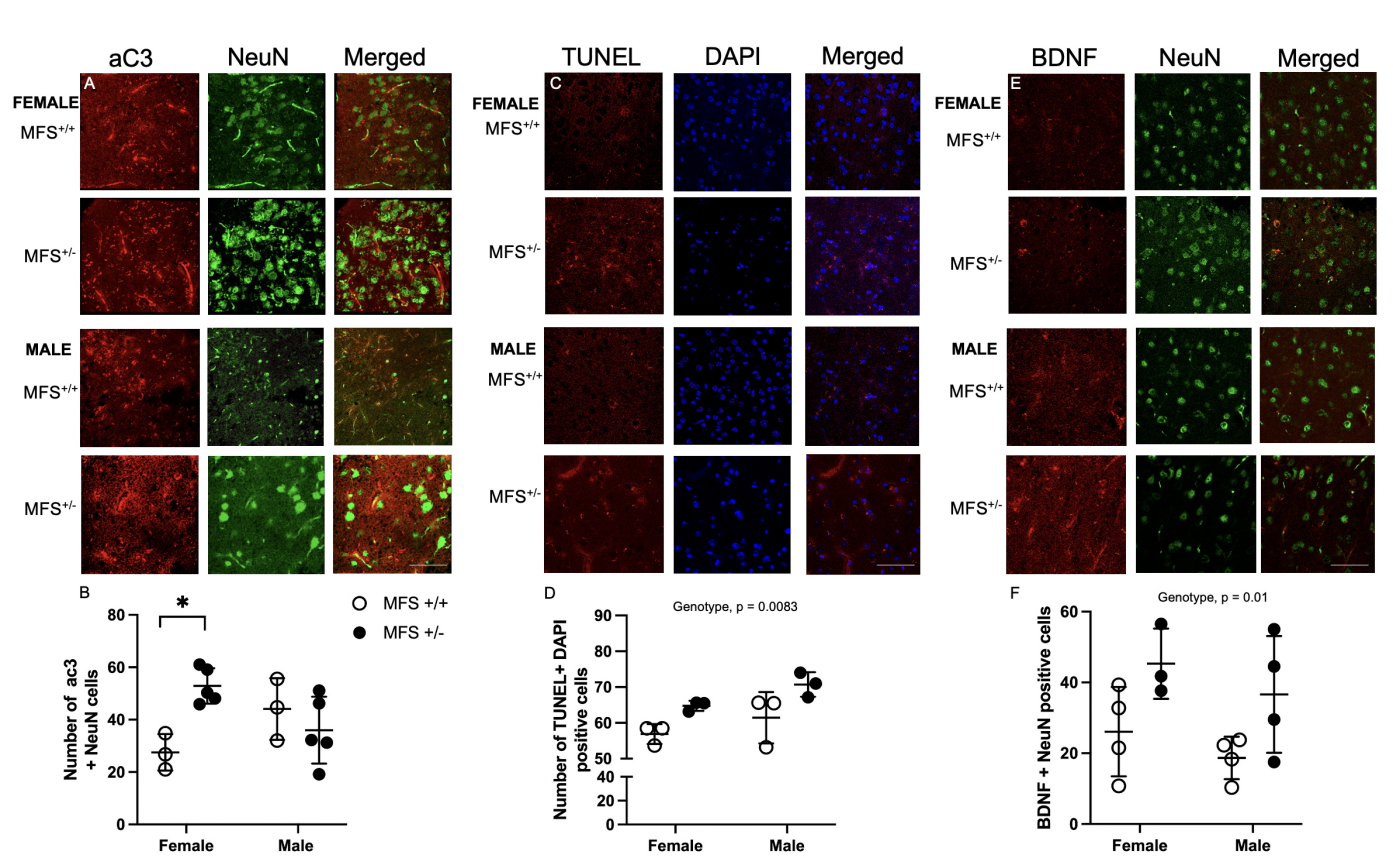
Active caspase-3 (aC3) and neuron nuclei (NeuN) in motor and sensory motor areas in male and female control and MFS mice. Representative images from female and male control and MFS mice **(A)** and quantification of aC3 and NeuN positive cells in brain cross sections from male and female control and MFS mice **(B)** . There was an increase in aC3 and NeuN-positive cells in MFS compared to age- and sex-matched controls (* *P* <0.05, Tukey’s pairwise comparison) **.** Assessment of Terminal deoxynucleotidyl transferase dUTP nick end labeling (TUNEL)-positive areas in motor and sensory motor areas in male and female control and MFS mice. Representative images from female and male control and MFS mice **(C),** and quantification of TUNEL and ′,6-diamidino-2-phenylindole (DAPI) positive cells in brain cross sections from male and female control and MFS mice **(D)** . There was an increase in TUNEL-positive cells in female and male MFS compared to controls ( *p* = 0.0083). Measurements of brain-derived neurotrophic factor (BDNF) and neuron nuclei (NeuN) expression in motor and sensory motor areas in male and female control and MFS mice. Representative images of female and male mice **(E)** and quantification of BDNF and neuronal nuclei (NeuN) positive cells in both male and female control and MFS mice **(F)** . There was an increase in BDNF neurons in female and male MFS compared to age- and sex-matched control animals ( *p* = 0.013).

## Description

Marfan syndrome (MFS) is a hereditary systemic disorder of the connective tissue caused by mutations in the gene encoding for the large glycoprotein fibrillin 1 (FBN1) that affects the cardiovascular, pulmonary, musculoskeletal, and ocular systems (Spencer, 2024). MFS can lead to manifestations such as aortic root aneurysm, scoliosis, pneumothorax, and lens dislocation, with aortic root dissection and rupture considered the most life-threatening complications (Milewicz et al., 2021).s

Despite recent advances in understanding the mechanisms involved in the development of MFS-associated aortapathy, there has been limited focus on the potential neurological and cerebrovascular implications. Previous clinical studies in MFS patients have revealed complications such as impaired cerebral blood flow, epilepsy, cephalgia, and varicose spinal veins, with reported increases in chronic migraine, neurodivergence, and intracranial aneurysms and stroke (Hofman et al., 1988; J. H. Kim et al., 2021; S. T. Kim et al., 2016, 2018; Spinardi et al., 2020). We have also recently reported a significant decrease in cerebral blood flow (CBF) in the mouse model of MFS ( *FBN1 ^C1041G/+^* ) (Curry-Koski et al., 2024), underscoring the potential impact of FBN1 abnormalities on cerebrovascular and brain function. In our previous work, we also demonstrated that by 6 months of age, both male and female MFS mice exhibited significant increases in blood-brain barrier (BBB) permeability and microglial activation, along with reduced microvascular density in the hippocampus (Curry-Koski et al., 2024). Notably, these changes mirrored those typically observed in 12-month-old healthy control mice, suggesting that MFS mice exhibit features indicative of a premature brain aging phenotype. BBB permeability changes in MFS mice have also been reported by other groups (Van der Donckt et al., 2015). It is well established that reduced CBF is linked to cognitive decline in patients with dementia (Weijs et al., 2023). The middle cerebral artery of MFS mice was thicker and had increased levels of reactive oxygen species (Onetti et al., 2016). Other studies have reported that reduced CBF can lead to hypoxia and nutrient deprivation, triggering oxidative stress, inflammation, cellular stress response, and apoptosis, resulting in neural damage and cell death (Claassen et al., 2021). Apoptosis, or programmed cell death, is a tightly regulated process essential for brain homeostasis, including neural network formation, removing damaged cells and proteins, and responding to brain injuries (Martin, 2001). Others have reported changes in neuroinflammation in MFS mice, as well as breakdown of the extracellular matrix (Manich et al., 2025). However, dysregulation of this important corrective mechanism can lead to various brain pathologies, including neurodegeneration and impaired recovery from injury (Manich et al., 2025; Moujalled et al., 2021; Radi et al., 2014).

In this study, we assessed the levels of aC3 and NeuN-positive neuronal cells within the cortical tissue of male and female control and MFS mice. Immunohistochemical analysis revealed a significant increase in the number of cleaved active caspase-3 (aC3) and NeuN-positive neuronal cells in the brains of female MFS mice compared to wild-type controls (p = 0.02), indicating elevated levels of neuronal apoptosis in this group ( **Figure 1A** ). In contrast, male MFS mice did not show a significant difference in aC3 and NeuN co-localization relative to their wild-type counterparts ( **Figure 1A** ). These findings suggest a sex-dependent vulnerability to neuronal cell death in the MFS mouse model ( **Figure 1B** , F ( _1, 12_ ) = 10.38, p = 0.0073, interaction between sex and genotype). There was no main effect of sex (p = 0.97) or genotype (p = 0.121).

To further assess apoptotic activity, we evaluated the colocalization of TUNEL and DAPI staining in cortical brain sections. Representative images are shown in **Figure 1C** . Quantitative analysis demonstrated a significant overall increase in apoptotic nuclei in MFS mice compared to wildtype controls ( **Figure 1D** ; F( _1,11_ ) = 8.78, *p* = 0.013). No main sex differences (p = 0.23) or interaction effects (p = 0.92) were observed.

To measure neuronal plasticity within cortical tissue we stained MFS brain tissue with antibody against BDNF and NeuN (Madinier et al., 2013). Representative images from female and male control and MFS mice are shown in **Figure 1F** . There were increased cell counts of neuronal BDNF in brain tissue in both female and male MFS brain tissue compared to age-matched wildtype littermates ( **Figure 1G** , F ( _1, 11_ ) = 8.782, p = 0.012). There were no main differences in sex (p = 0.225) or interaction between sex and genotype (p = 0.922)

Clinical studies have reported that individuals with MFS have increased risk for cerebrovascular alterations due to their vascular tortuosity phenotype (Parlapiano et al., 2020). A recent study investigating the neuropathology in the same MFS mouse model showed a systemic pre-mature aging phenotype is present in the six-month old mice (Curry-Koski et al., 2024). Specifically, the researchers reported reductions in blood flow of the posterior cerebral artery, as well as increased blood-brain permeability and neuroinflammation (Curry-Koski et al., 2024). The present study has added to the neurological phenotype of the MFS. Within the sensory and motor cortex, MFS mice showed increased levels of apoptotic neurons compared to wildtype animals along with increased levels of plasticity.

Interestingly, neuronal aC3 levels were significantly increased in females compared to males; this is an interesting sex-dependent phenotype in the mouse model of MFS. Clinically, mortality as a result of MFS affects males and females at an equal rate (Nucera et al., 2022). However, sex differences in related outcomes have been reported in clinical and preclinical studies (Sakai & Backer, 2023). A possible explanation for the observed sex difference in our present study might be the age of the mice, estrogen has been shown to be neuroprotective in brain response to stress. Furthermore, these findings raise the possibility of an estrogen receptor–mediated mechanism contributing to the observed sex difference, though the direction of this effect may vary depending on context, age, and underlying disease pathology (Saddic et al., 2023; Waters & Simerly, 2009).

Together with our recently publication (Curry-Koski et al., 2024), our findings reveal an increased vulnerability to neurodegeneration in the cortical tissue of both male and female MFS mice at 6 months of age. This pilot study is the first to characterize neurodegenerative processes in the well-established MFS mouse model. Notably, we observed elevated levels of neural plasticity in sexes, which may be associated with the increased apoptotic activity. These novel insights into the neural pathology of MFS underscore the need for further research to delineate the temporal progression of neurodegeneration and to explore additional pathological markers involved in programmed cell death and neural plasticity pathways in the mouse model of MFS **.**

## Methods

The mouse model used in the study carries a heterozygous missense mutation in the *FBN1* gene ( *FBN1 ^C1041G/+^* , cysteine to glycine substitution Cys ^1041^ →Gly), recapitulating the aortic aneurysm phenotype observed in MFS patients (Cook et al., 2010; Marque et al., 2001). A breeding colony was established by crossing male MFS ( *FBN1 ^C1041G/+^* ) with female control ( *FBN1 ^+/+^* ) mice obtained from The Jackson Laboratory (Bar Harbor, ME). Male and female mice were housed in the institutional animal facility with standard animal room conditions (25 °C, 12-hour light-dark cycles, ≤5 mice in a cage). All animals used in this study were cared for in compliance with the Guide for the Care and Use of Laboratory Animals and all experimental procedures were performed according to the Midwestern University Institutional Animal Care & Use Committee (IACUC) approved protocol (AZ-2936). The experiments were reported according to the ARRIVE guidelines. At 6 months of age, male and female MFS, and control littermates (n = 4-6/group) were euthanized using 5% isoflurane (confirmation by pedal reflex), followed by cervical dislocation.

***Immunofluorescence of Brain Tissue***

Male and female mice brain tissue was removed and fixed in 4% paraformaldehyde (PFA) for 24 hours, cryo-sectioned at 20µM thickness and serially mounted onto slides. Microscope slides were stored at -80°C removed prior to starting immunofluorescence experiments. To investigate neurodegeneration and plasticity, immunofluorescence analysis of brain tissue was performed. To assess apoptosis-associated neurodegeneration cortical brain sections were stained with active capase-3 primary antibodies (AC3, 1:100, Cell Signaling Technologies, catalog # 9662). All brain sections were also stained with a marker for neuronal nuclei (NeuN, 1:200, Abcam, catalog # ab177487). We measured plasticity using brain-derived neurotrophic factor (BDNF, 1:100, Abcam, catalog # ab108319). Brain sections were incubated overnight at 4˚C with primary antibodies diluted in 0.5% Triton X, and with secondary antibodies Alexa Fluor 488 or 555 (1:200, Cell Signaling Technologies, catalog# 4408 and 4413) at room temperature for 2 hours, followed by staining with 4’, 6-diamidino-2 phenylindole (DAPI, 1:10000, Fisher Scientific, catalog # EN62248). Microscope slides were cover-slipped with Fluro-mount mounting media (Fisher Scientific, catalog # OB100-01) and stored at 4°C until imaging.

***Terminal deoxynucleotidyl transferase (TdT)-mediated dUTP nick end labeling (TUNEL)***

Brain tissue from animals was stained using TUNEL reactions, which were performed as described according to the manufacturer’s instructions with some modifications (Fisher Scientific, catalog # 501964491). Briefly, tissue was incubated with 0.5% TritonX for 5 minutes at room temperature. After rinsing tissue with PBS, sections were incubated in TUNEL Equilibration buffer for five minutes at room temperature and then immediately after TUNEL reaction mixture (TdT and fluorescein-labeled nucleotide mixture) was added to brain tissue for 2 hours at room temperature. Tissue was rinsed with PBS and then incubated with DAPI (1:10000, Fisher Scientific, catalog # EN62248) for 5 minutes. Tissue was rinsed with PBS and slides were cover slipped with (Fisher Scientific, catalog # OB100-01) and stored at 4°C until imaging.

***Imaging & Statistical Analysis***

Stained cortical brain sections were imaged using a Leica TCS SPE confocal microscope to create z-stacks. A minimum of 2 cortical brain sections were included for analysis of each animal. For analysis of confocal imaging, two observers, who were blinded to experimental conditions, completed cell count colocalization of active caspase-3 and NeuN, BDNF and NeuN, and finally, TUNEL and DAPI analysis using ImageJ (NIH). The cell counts for each animal were averaged for final analysis.

GraphPad Prism 10.02 was used to analyze immunofluorescence staining measurements. In the GraphPad Prism, D’Agostino-Pearson normality test was performed prior to two-way ANOVA analysis, when comparing the mean measurement of both sex and genotype groups for immunofluorescence staining. Significant main effects of two-way ANOVAs were followed up with Tukey’s post-hoc test to adjust for multiple comparisons. Data were checked for statistical outliers by using the Grubbs' test. All data are presented as mean + Standard Deviation (SD). Statistical tests were performed using a significant level of *P* ≤ 0.05.
